# Mast Cells as Potential Accelerators of Human Atherosclerosis—From Early to Late Lesions

**DOI:** 10.3390/ijms20184479

**Published:** 2019-09-11

**Authors:** Petri T. Kovanen

**Affiliations:** Wihuri Research Institute, Biomedicum Helsinki 1, 00290 Helsinki, Finland; Petri.Kovanen@wri.fi

**Keywords:** atherosclerosis, cardiovascular disease, mast cells

## Abstract

Mast cells are present in atherosclerotic lesions throughout their progression. The process of atherogenesis itself is characterized by infiltration and retention of cholesterol-containing blood-derived low-density lipoprotein (LDL) particles in the intimal layer of the arterial wall, where the particles become modified and ingested by macrophages, resulting in the formation of cholesterol-filled foam cells. Provided the blood-derived high-density lipoproteins (HDL) particles are able to efficiently carry cholesterol from the foam cells back to the circulation, the early lesions may stay stable or even disappear. However, the modified LDL particles also trigger a permanent local inflammatory reaction characterized by the presence of activated macrophages, T cells, and mast cells, which drive lesion progression. Then, the HDL particles become modified and unable to remove cholesterol from the foam cells. Ultimately, the aging foam cells die and form a necrotic lipid core. In such advanced lesions, the lipid core is separated from the circulating blood by a collagenous cap, which may become thin and fragile and susceptible to rupture, so causing an acute atherothrombotic event. Regarding the potential contribution of mast cells in the initiation and progression of atherosclerotic lesions, immunohistochemical studies in autopsied human subjects and studies in cell culture systems and in atherosclerotic mouse models have collectively provided evidence that the compounds released by activated mast cells may promote atherogenesis at various steps along the path of lesion development. This review focuses on the presence of activated mast cells in human atherosclerotic lesions. Moreover, some of the molecular mechanisms potentially governing activation and effector functions of mast cells in such lesions are presented and discussed.

## 1. Experimental Atherosclerosis: from Mouse to Mechanisms and Proofs of Concept—Still Far from Perfection

Atherosclerosis is a progressive inflammatory disease which is characterized by accumulation of blood-derived lipids in the arterial wall, and, as a consequence of it, a long-lasting process ensues, in which local lesions are formed as a result of a series of highly specific cellular and molecular responses [1,2,3]. The above definition of human atherosclerosis as a lipid-driven disease perfectly matches with the animal models used for atherosclerosis research already for a period of almost one hundred years. Thus, as a rule, the experimental animals are fed a cholesterol-rich Western-type diet, which causes strong elevation of plasma lipid levels, which, again, continues to induce lipid-containing atherosclerotic lesions in the susceptible areas of the arterial tree. Traditionally, rodents (rabbits, rats, hamsters, and guinea pigs), swine, and non-human primates have been used as animal models of atherosclerosis [4,5].

In 1992, a dramatic change in the field of experimental atherosclerosis studies occurred, as the first line of genetically engineered knockout animal models, namely, the apolipoprotein E knock-out (*Apoe^-/-^*) mice, was published by two groups of scientists [6,7]. Such mice developed spontaneous hypercholesterolemia and arterial lesions [8]. Ever since an increasing number of knockout/transgenic animal models have been generated, a common feature of all models having been a genetically determined hypercholesterolemia, i.e., high plasma cholesterol level since birth. Of note, mice lacking low-density lipoprotein receptors, the *Ldlr^-/-^*mice, are counterparts to the severe homozygous form of human disease familial hypercholesterolemia (FH), and, like the patients with homozygous FH, the mice show strong increase in the concentration of low-density lipoprotein (LDL) cholesterol [9]. Such mice show an early development of atherosclerotic lesions in the arterial tree, and, moreover, an accumulation of cholesterol in the skin, particularly, when the mice are also fed a cholesterol-rich high-fat diet.

Despite the above-described progress in creating animal models of human atherosclerosis, a great challenge has been the lack of spontaneous atherothrombotic events, i.e., absence of the very final acute stages of atherosclerosis, as they occur in humans. Importantly, however, cross-breeding *Apoe^-/-^* mice with mice containing a heterozygous mutation of the fibrillin-1 gene (equivalent to the Marfan syndrome) has generated mice which show many features of human end-stage atherosclerosis, resulting in plaque rupture, myocardial infarction, and sudden death of the mice [5]. Thus, at present, the various genetically engineered mouse models as a whole provide suitable tools to get insight into the mechanisms of various stages of atherogenesis, such as they occur in humans.

The genetically engineered mice as models of atherosclerosis have provided invaluable tools to examine the roles of mast cells in atherogenesis *in vivo*. Thus, by using *Ldlr^-/-^* mice which had been crossbred with mast cell-deficient *Kit^W-sh/W-sh^* mice, i.e., *Ldlr^-/-^Kit^W-sh/W-sh^* double knockout mice , Sun and colleagues demonstrated that mast cells promote atherosclerosis by releasing pro-inflammatory cytokines (IL-6 and IFN-γ) [10]. Moreover, in the same year, Bot and colleagues demonstrated in *Apoe^-/-^* mice that targeted activation of perivascular mast cells in an atherosclerotic carotid arterial segment promotes atherogenesis and, most importantly, also induces plaque destabilization [11]. These two seminal pieces of work provided the first proof of the concept of a mast cell—atherosclerosis axis.

This review will revolve around on the presence activated mast cells in human atherosclerotic lesions. Also, some selected molecular mechanisms potentially governing activation and effector functions of mast cells in the human lesions are briefly discussed.

## 2. Atherogenesis—A Brief Outline of a Long Path of Events

Infiltration of circulating apolipoprotein(apo) B-containing lipoproteins, notably of the apoB-100—containing LDL particles, into the inner layer, the intima, of atherosclerosis-susceptible segments of the arterial tree is the root cause of atherogenesis ([12]. Because of the prolonged residence time in the intima, the LDL-particles, whether proteoglycan-bound or free-floating in the intimal extracellular fluid, are susceptible to modification by intimal proteases, lipases, or oxidizing agents [13,14,15]. The extracellular modifications of the infiltrated LDL-particles initiate local innate and adaptive immune responses in the intima, the modified lipid components of the particles possessing particularly strong proinflammatory properties [16,17]. Thus, for example, even the bioactive lipids generated in minimally oxidized LDL-particles induce chemotaxis and endothelial adhesion of circulating monocytes to endothelial cells [18]. The monocytes then enter the intima and differentiate into macrophages, so generating an even-growing population of intimal macrophages. Regarding the “big bang” initiation of atherosclerosis, it is not possible to decide which comes first—the circulating monocytes or the circulating LDL-particles. Rather, they may start entering the atherosclerosis-susceptible sites of the arterial tree in parallel.

As the monocytes are transformed into macrophages, they also begin to express scavenger receptors, which are able to recognize the modified LDL particles and then to ingest them [19]. Uptake of the modified cholesterol-containing LDL particles leads to accumulation in the macrophages of cholesterol as cholesteryl ester-containing cytoplasmic lipid droplets, thereby making the cells appear “foamy”. The emergence of foam cells is the first sign of incipient atherogenesis, and thereby presents the typical histological hallmarks of early atherosclerosis, the fatty streak stage [20].

A gradual accumulation of cholesterol in the intima is a fundamental process in the progression of atherosclerosis. Actually, there is no atherosclerosis without intimal cholesterol accumulation, as stated by the founder of the lipid hypothesis of atherosclerosis Nikolai Anitschkow already more than 100 years ago [21]. Since cholesterol cannot be degraded into its building blocks, it must be removed from the intima in order not to accumulate there. The plasma apolipoprotein A (apoA)-containing high-density lipoprotein (HDL) particles which have entered the intimal space perform this critical anti-atherosclerotic function. Particularly, a subfraction of HDL, the pre-beta HDL particles, bind to ABCA1 receptors on macrophage surfaces, and efficiently take up cholesterol from them [22,23]. Since the HDL particles do not directly accept LDL cholesterol, the only way for intimal LDL cholesterol to return back to the circulating blood is via macrophages. Thus, the intimal macrophages are functioning as essential intermediaries in this from LDL to HDL transfer of cholesterol. However, since the intimal macrophages become cholesterol-filled foam cells, it appears that the HDL-dependent induction of cholesterol efflux from macrophages is continually dysfunctional, i.e., less cholesterol is released than taken up [24,25].

During such imbalance between cholesterol uptake and release by macrophages, cholesterol keeps accumulating as cytoplasmic cholesteryl ester droplets, until the cytoplasm is filled with the droplets, i.e., foam cells are formed. And even worse, the lipid droplets are metabolically active and constitute a futile ATP-wasting energy-consuming cycle of continual hydrolysis of cholesteryl esters and re-esterification of the liberated cholesterol, and by time may prepone the death of the ageing foam cells [26]. If the apoptotic foam cells are not effectively efferocytosed by their neighboring macrophages, then they undergo post-apopotic secondary necrosis, whereupon the cytoplasmic cholesteryl ester droplets become displaced into the extracellular space of the intima, and, together with the remains of the dead macrophages form a necrotic lipid core [3,27,28].

The appearance and growth of a necrotic lipid core heralds the generation of an advanced atherosclerotic lesion, i.e., a conversion of a fatty streak into an atherosclerotic plaque or atheroma [29]. In an atheroma, the necrotic lipid core is separated from the passing blood by a protective fibrous cap, in which the smooth muscle cells of a “synthesizing phenotype” produce the collagen fibers necessary to stabilize the plaque. Thus, an advanced atherosclerotic lesion is a composite of lipids and fibrotic tissue, and, depending on which of the components dominates, the lesion is called a fibrous, fibro-fatty, or fatty plaque. Of note, in this terminology “fatty” refers to the macroscopically visible fat, which is composed of the extracellular lipid core.

The atherosclerotic cardiovascular diseases, particularly coronary artery disease, remains silent until the arterial blood flow becomes compromised and causes ischemic symptoms—either chronically due to slowly narrowing of the arterial lumen, or acutely due to a thrombotic occlusion of the lumen [30]. The growth of an atheroma, particularly of a fibro-atheroma, typically causes stenotic obstruction of the blood flow with ensuing turbulence of the flow. The flow disturbance causes endothelial dysfunction, and if further surface damage occurs due to metabolic disturbances or immune insults, the endothelial cells may become detached from the basement membrane, and so expose the blood to a prothrombotic surface with ensuing local platelet-rich thrombus formation [31]. When compared with the superficial plaque erosions, deeper plaque fissures due to rupture of a thin fibrous cap are usually more dramatic, as they allow the blood to become in contact with the exceedingly prothrombotic necrotic lipid core, so enhancing the likelihood of an extensive thrombus growth with ensuing full occlusion of the coronary artery lumen [30].

Clinically the most significant and dangerous process in coronary atherosclerosis is the development of a fatty plaque, which often grows outward and therefore fails cause chronic ischemic symptoms [32]. Such non-obstructing lesions are characterized by an ever-growing necrotic lipid core accompanied with an ever-thinning fibrous cap, and they cause atherothrombotic complications often without warning [33].

Taken together, intimal infiltration and accumulation of atherogenic plasma lipoproteins, notably of LDL particles, and infiltration of monocytes and their conversion into macrophages are the driving forces of atherosclerotic lesion growth. The local process within the intima is a low-degree chronic “atheroinflammation”, and the executors of the inflammation are inflammatory cells of three types, notably the macrophages, T cells, and mast cells [34]. Vast arrays of subtypes of both macrophages and T cells have been characterized in atherosclerotic lesions [3,35,36,37]. In this review, the possible roles and relevance of mast cells in the pathogenesis of atherosclerosis, as briefly outlined above, will be presented and discussed.

## 3. Mast Cells and Their Presence in Human Atherosclerotic Lesions

### 3.1. General Definition of Mast Cells and Their Functions

Mast cells are tissue-dwelling immune cells of hematopoietic origin, which have a widespread distribution in nearly all tissues, but are typically most abundant at host-environment interfaces, such as the skin and various mucosal surfaces. Thus, mast cells are ideally situated to act during the first line defense against external pathogens and other environmental insults [38]. Because of their high reactivity to external allergens, the mast cells are canonically viewed as primary effector cells in allergic disorders [39]. The potential roles of mast cells in the connective tissue-rich internal organs have received less attention, but, more recently, mast cells have become known for their detrimental actions in several local or systemic diseases such as rheumatoid arthritis, cancer, and cardiometabolic diseases [40,41,42,43]. Importantly, mast cells are currently emerging as multifunctional effector cells not only in several pathological but also physiological clinical conditions [44]. In the tissues, mast cells are usually found in close proximity to epithelial cells, endothelial cells, and nerves [45], and this also applies to cardiac mast cells, being located in the myocardium or in the epicardial coronary arteries. Rodent mast cells, which contain a vast armamentarium of proteases in their granules have been traditionally classified into connective-tissue mast cells and mucosal mast cells due to their location, while human mast cells, based on their more restricted protease expression pattern, have been classified into those containing both tryptase and chymase, and into those containing only one of the two granule neutral serine proteases, namely tryptase [46,47]. However, it is important to note that the human mast cells constitute a highly heterogenous population of cells with differences in surface receptors and mediator content [48]. Moreover, like macrophages and neutrophils, the mast cells show phenotypic and functional plasticity [49].

### 3.2. The Abdominal Aorta

Historical analysis of the early findings demonstrating the presence of mast cells in the arterial walls is beyond the scope of this review. The reader is advised to consult excellent reviews on the topic, in which the presence and potential roles of histamine and heparin derived from the mast cells present the in the arterial wall of human beings and experimental animals are discussed with a particular focus on atherosclerosis research [50,51].

More recently, evidence for the presence of mast cells in the atherosclerotic human aortic wall was presented independently by two groups [52,53]. Atkinson and coworkers studied autopsied young subjects aged 15 to 34 years, who had in their aortas and coronary arteries both healthy segments and segments containing raised lesions, which were classified as fatty streaks, fibrofatty plaques, and fibrous plaques. Overall, they found that mast cells were located primarily in the outer arterial layer or adventitia, although occasional mast cells were seen in the bordering media and in the intima. Surprisingly, the numbers of mast cells in the adventitia were reported to approach those observed in histologic sections of skin in patients with mastocytosis, a disease in which very high numbers of mast cells accumulate particularly in the skin [54]. Moreover, mast cell numbers in the segments with advanced lesions had about twice as many mast cells as the segments without lesions, the greatest numbers of mast cells being found in segments with lipid-containing lesions (fatty streaks and fibro-fatty plaques). Moreover, in that study, more mast cells were found in the samples derived from the dorsal than from the ventral half of the aortic segments. Since the dorsal half of the aortic wall was known to be more lesion-prone than the ventral half, Atkinson and coworkers rightly suggested that adventitial mast cells may potentially play a role in the initiation and evolution of atherosclerotic lesions in the aorta.

In our laboratory, Dr. Kaartinen focused on mast cells in the intimal layer of the abdominal aortic wall [53]. Specimens of normal and atherosclerotic aortic segments from 35 autopsies of persons ranging from 13 to 67 years old were stained with monoclonal antibodies against the two major proteases of mast cells, tryptase and chymase. As expected, like the mast cells in all human tissues, the mast cells in the aortic intima contained tryptase, and, moreover, a highly variable number of them also contained chymase. Thus, in the normal intima, the fatty streaks, and the atheromas, the number of chymase-containing mast cells ranged from 0 to 100%, the average proportion of chymase-containing mast cells of the total number of mast cells being about 40% in each lesion type. Thus, mast cells of two phenotypes differing in their neutral protease composition were found in the human aortic intima, the expression of chymase being highly individual. However, no association between the mast cell phenotype, i.e., chymase-positive versus chymase-negative, and lesion severity could be observed. Disappointingly, in this particular study, the most advanced lesions, the atheromas, contained far less mast cells (3/mm^2^) than did the normal intima (15/mm^2^) or the fatty streaks (15/mm^2^). A closer analysis of the regional distribution of mast cells in the atheromas revealed, however, a highly uneven distribution of the mast cells in the various parts of the atheromas. Thus, while no mast cells were present in the necrotic lipid core, on average, 1 and 8 mast cells per mm^2^ were observed in the fibrous cap and the vulnerable shoulder regions, respectively.

Although the numbers of mast cells reported in the above-cited study were low when compared with other types of inflammatory cells in the atheromas, the key finding demonstrating that the vulnerable shoulder regions of the atheromas contained relatively the highest distribution density of mast cells among all the regions in the atheromas strongly suggested that mast cells may potentially play a role also in the late stages of atherosclerosis when the vulnerable shoulder regions develop as parts of a raised lesion. This particular finding was actually the starting signal for further studies, in which we focused on atherosclerotic coronary arteries of patients who had succumbed to an acute atherothrombotic event with ensuing myocardial infarction. In an infarct-related coronary artery, often many anatomically remote complex plaques are found, and, moreover, between them lesions displaying all stages of atherogenesis ranging from a near-normal intima to the ruptured culprit plaque, also exist [55]. Thus, even a single atherosclerotic human coronary artery obtained at autopsy from a patient who died of myocardial infarction allowed us to evaluate the potential role of mast cells in various stages of atherogenesis, as will be described below.

### 3.3. Epicardial Coronary Arteries

**Figure 1:**
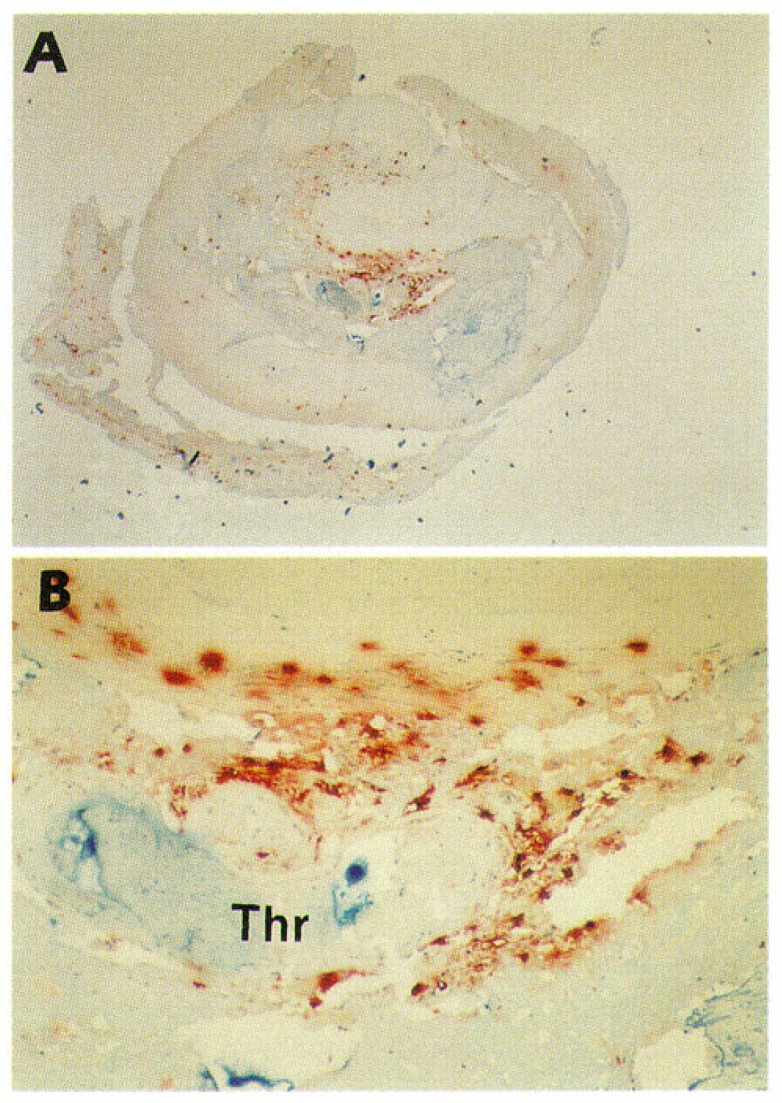
Mast cells in an advanced atherosclerotic plaque of a highly atherosclerotic human coronary artery. This is a histological cross section of an eroded coronary segment with thrombotic occlusion. The specimen was from a 78-year-old man who died of myocardial infarction 3 h after the onset of symptoms of an acute coronary event. Mast cells were stained with monoclonal antibody G3 for the mast cell-specific neutral serine protease tryptase (red-brown). (**A**) Original magnification ×20. (**B**) Detail of the erosion site; original magnification × 200. “Thr” indicates thrombus. Numerous macrophages and T lymphocytes also accumulated in this area (not stained in this particular section). Reproduced from Kovanen PT, Kaartinen M, Paavonen T. Infiltrates of activated mast cells at the site of coronary atheromatous erosion or rupture in myocardial infarction [57].

Like in the human aorta, in the myocardial infarct-related human coronary artery mast cells accumulate during the progression of atherosclerosis [56]. Overall, the anatomic distributions of mast cells in coronary lesions were found to be similar to those in aortic lesions, the numbers being highest in the vulnerable shoulder regions, the predilection sites of plaque rupture. However, the increases in mast cell numbers in the progressing coronary atherosclerotic lesions were found to be much stronger than those in aortic lesions. Most importantly, light and electron microscopic studies of mast cells in the shoulder regions revealed degranulation of mast cells as a sign of their activation. The proportion of activated mast cells was much higher (50-fold) in the shoulder region than in the normal intima, suggesting that they had actively participated in the destabilization and ensuing rupture of the coronary atheromas, and so may have contributed to the triggering of the acute coronary events. This expectation received strong support from the observation that in patients who had died of acute myocardial infarction, up to 200-fold more activated mast cells were present at the actual site of atheromatous erosion (endothelial desquamation) or rupture in the culprit lesion than in the unaffected coronary intima of the same culprit coronary artery [57]. Figure 1 shows immunostaining of mast cells in a highly atherosclerotic human coronary artery. In this coronary segment, the endothelial layer was eroded, and the severely narrowed lumen was totally occluded with a thrombus. Accumulations of mast cells (red-brown) can be clearly discerned. Numerous macrophages and T lymphocytes had also accumulated in this area (not shown).

However, the causal relationship between activation of mast cells in vulnerable coronary plaques and in already ruptured plaques has been questioned. Thus, one may ask a critical question whether mast cell activation is a cause or consequence of an acute coronary event. Although no definitive answer to this question can be given in humans, the following findings support the notion that mast cells are activated before the actual event. First, in ruptured plaques, not only the number of mast cells but also the degree of mast cell degranulation is highest at those sites where the numbers of other inflammatory cells are highest [57]. Moreover, in atherectomized coronary samples obtained from patients with unstable angina pectoris, the lesional mast cells were shown to be accompanied by T cells and macrophages, and, the more severe were the symptoms of angina pectoris, the greater were also the numbers of the T cells surrounding the mast cells [58]. Moreover, the Human Leukocyte Antigen—DR Isotype (HLA-DR) expression in the T cells and macrophages was increased, revealing that the T cells and macrophages were also in an activated state. Thus, in addition to mast cells, the T cells and macrophages in the infiltrates of inflammatory lesions also showed signs of activation, which supports the idea that this entire triad of inflammatory cells plays a primary, i.e., causative role in the final atherothrombotic events of atherosclerotic coronary artery disease.

## 4. Grading Mast Cell Activation in Human Atherosclerotic Coronary Arteries

In the human arterial wall, like in other human tissues, histological determination of mast cell activation depends on microscopic observation of extracellular granules in the immediate vicinity of a mast cell. Among all tissue resident cells, only mast cells produce heparin and tryptase, and then pack them densely together in cytoplasmic granules, which allows completely reliable and specific detection of mast cells in tissues. Surprisingly, however, in our hands the immunocytochemical staining for mast cell granule heparin proteoglycans yielded paradoxical results which indicated almost a complete loss of mast cells in the shoulder areas of aortic atheromas, i.e., the areas which were later shown by immunohistochemical detection of tryptase to contain most of the mast cells in coronary atheromas, as discussed above [53,56,57]. The explanation for this discrepancy appears to lie in the different sensitivities of the two methods. Since mast cell degranulation leads to a variable loss of the heparin- and tryptase-containing cytoplasmic secretory granules from the activated mast cells, the insensitive heparin staining failed to detect excessively stimulated mast cells which had lost a significant proportion of the heparin they had contained. In contrast, the sensitive immunohistochemical staining of tryptase must have detected even the remaining traces of tryptase, and so provided a tool for accurate determination of the actual mast cell numbers irrespective their state of activation.

Another advantage of the sensitive immunostaining for tryptase is its ability to detect at high magnification exocytosed mast cell granules, i.e., those residing extracellularly in the near vicinity of a mast cell, so allowing one to detect and quantify the mast cells which have been activated to degranulate [57]. Since a fraction of the heparin proteoglycans and of proteoglycan-complexed tryptase appears to be lost from the exocytosed granules, any tryptase-poor extracellular “granule remnants” particularly need the sensitive tryptase immunostaining for their detection [59,60]. Since the total number of mast cells and the number granules around each mast cell can be counted, not only quantification of the proportion of degranulated mast cells but also the degree of degranulation of an individual mast cell is possible. Applying the sensitive tryptase-staining method for adventitial mast cells in the culprit coronary arteries of patients who had died of myocardial infarction, we could observe that the total number of mast cells present in the adventitia backing ruptured plaques was higher (on average about 100 mast cell/mm^2^) than that in the adventitia backing non-ruptured plaques (40 mast cells/mm^2^), which, again, was higher than that in the adventitia backing normal intima (20 mast cells/mm^2^) [61]. These high numbers of mast cells in the coronary adventitial layer were similar to those obtained earlier for the adventitial mast cells in the human abdominal aortas by Atkinson and coworkers [52].

The most relevant finding in our coronary death study was that the percentage of adventitial mast cells with extensive degranulation (defined as 5 extracellular granules in the section plane) was significantly higher in the segments with plaque rupture (about 50%) than in those with non-ruptured plaque (17%) or in segments with normal intima (11%) [61]. These observations on mast cell number and activation strongly supported the general concept of the inflammatory cells present in the adventitia actively participating in the pathogenesis of atherosclerosis, even from the early stages on [62]. Recent studies in many laboratories suggest an important role also for the coronary perivascular adipose tissue as a source of inflammatory mediators in atherosclerotic coronary artery disease and vasospastic angina [63,64]. Inasmuch as mast cells are known to be present in various adipose tissue depots in human subjects and to participate in chronic low-grade inflammation [43,65], studies about the proinflammatory role of mast cells in the pericoronary adipose tissue will be of great interest.

## 5. Potential Mast Cell Triggers in Human Atherosclerotic Coronary Arteries

As presented above, microscopic observations in human atherosclerotic coronary arteries have demonstrated the presence of degranulated mast cells in the lesions, so providing firm evidence for the presence of mast cell-activating principles in the diseased arteries. What may then have triggered the mast cells to degranulate in the lesions? A clear-cut demonstration of increased numbers of extracellular granules around tissue mast cells has been generally considered to reflect an allergic or anaphylactic, i.e., immunoglobulin E (IgE)-dependent activation of mast cells [39,48,66]. Thus, we can consider IgE as a possible activator of mast cells in atherosclerotic lesions. However, the only clear-cut clinical example of a potentially IgE-mediated activation of human coronary mast cells is the allergic angina or Kounis syndrome, defined as the co-incidental occurrence of an acute coronary syndrome with a hypersensitivity reaction [67]. Mechanistically, the IgE-mediated mast cell degranulation with ensuing local histamine and protease release leads to coronary spasm of an atherosclerotic segment of a coronary artery, and even to an atherothrombotic coronary event due to plaque erosion or rupture. This hypersensitivity disorder of the atherosclerotic coronary arteries may present itself as an allergic angina, allergic myocardial infarction, or stent thrombosis, in which the coronary wall adjacent to a stent is infiltrated with mast cells and/or eosinophils [68]. Very recently, Niccoli and coworkers extensively reviewed the potential roles of effector cells of allergic inflammation, i.e., mast cells, eosinophils, and basophils, in the context of coronary artery disease progression and instability, and in the occurrence of adverse events after stent implantation [69]. As will be discussed later, also non-classical IgE-mediated (superantigenic) activation of human myocardial mast cells has been demonstrated [70].

In the above cited studies in which we found degranulated, i.e., activated mast cells in atherosclerotic coronary arteries of autopsied patients, there were no suspicions of allergy-related deaths [56,57]. Thus, we are inclined to reject the hypothesis of an allergic inflammation being a component of evolving human coronary atherogenesis, and, rather need to consider stimuli which do not trigger the classical antigen-specific IgE-mediated mast cell activation with ensuing degranulation. Indeed, recent work has identified a vast number of mast cell-activating factors which trigger differential release of newly synthesized proteinaceous and lipid mediators from the activated mast cells. Such stimulus-specific secretory pathways usually involve exocytosis of the cytoplasmic secretory granules, as well, and may result from mast-cell activation by e.g., adrenomedullin, complement factors, defensins, endothelin, immunoglobulins of the IgG class, hemokinin, nerve growth factor, sphingosine-1-phosphate, substance P, thrombin, IL-33, stem cell factor, Toll-like receptor ligands, and neuropeptides [44,48,71,72,73,74,75]. Of major interest are the findings demonstrating that human mast cells release the matrix metalloproteinase MMP-9 and cytokines on contact with activated T cells, and that mast cells can be activated by microparticles released from T cells [76,77] Considering that atheroinflammation involves both T cells and mast cells, such cell-cell interaction between activated T cells and mast cells may promote locally degradation of the extracellular and pericellular matrices, and so render the plaque unstable and susceptible to erosion or rupture.

However, only a few of the above-listed stimuli used in experimental systems have been tested for their ability to activate cultured non-transformed human mast cells, and, actually, many of the tested ones have failed to act as an effective stimulant of such mast cells. What are then the potentially relevant triggers of mast cells in an evolving human atherosclerotic lesion? Clearly, the minimum requirement is that the mediator (agonist) is present in the lesion and that the lesional mast cells express a receptor which is responsive to the agonist [43,44,75]. Of note, as noted above, most of the experimental cell culture results have been obtained using mast cells of non-human origin, or immortalized human mast cell mast cell lines, such as the HMC-1 and the LAD-2 cell lines, and so provide only suggestive evidence regarding their more physiological counterparts [78]. In our laboratory, we have performed experiments with cultured primary human mast cells [79]. Using these cells, we observed that, in contrast to some other laboratories, oxidized LDL particles, which are considered critical components of atherogenesis, do not trigger mast cell activation, whereas addition of immune complexes composed of oxidized LDL particles and IgG antibodies directed against LDL particles did trigger degranulation of the cultured mast cells [80]. However, a word of caution is appropriate. Thus, although we used human primary mast cells, such “physiological” mast cells were allowed to differentiate and grow in a culture medium whose composition contained only a few selected growth and differentiation factors known to be present in the human arterial intima. Thus, it is obvious that the growth and differentiation conditions did not even closely resemble those of the dynamically changing multicompartmental extracellular fluid present in a human atherosclerotic lesion. Moreover, no coculture experiments were performed, so precluding us to draw any conclusion from the complex cell-to-cell interactions present in such lesions. Thus, we have to conclude that, since the mast cells readily adapt their phenotype in response to changes in the microenvironmental factors [49], only isolation of lesional mast cells and examination of their sensitivity to the ligands present in the lesions will take us further in search for actual responsiveness of coronary mast cells to triggers relevant to human atherogenesis.

At present, among the candidate triggers of mast cells in advanced atherosclerotic human coronary lesions is the complement system [81]. Thus, in such lesions the complement system is activated with ensuing generation of the anaphylatoxins C3a and C5a, and, moreover, the lesional mast cells express the anaphylatoxin receptor C5aR, so rendering C5a a strong candidate of mast cell activation in human coronary plaques [82]. Indeed, C5a-mediated mast cell activation was shown to promote vein graft disease in apolipoprotein E-deficient mice by accelerating the formation of atherosclerotic lesions and plaque disruptions in the grafts [83] Importantly, substance P- and calcitonin gene-related peptide (CGRP)-containing sensory neurons are found in the adventitial layer of human coronary arteries (but not in the atherosclerotic lesions), where they are in close contact with mast cells backing the atherosclerotic lesions [84]. Accordingly, the mast cell-activating potential of these neuropeptides is restricted to the adventitial layer of a coronary artery. However, since the adventitial mast cells are located adjacent to *vasa vasorum* which grow across the medial layer into advanced coronary lesions forming there neovascular sprouts, the mast cell-derived soluble products can reach the contractile smooth muscle cells of the medial layer, and ultimately find their way to the lesions, where they can act locally [84]. Interestingly, strongly increased numbers of activated coronary adventitial mast cells have been found in a patient who suffered from the classic variant form of angina pectoris, i.e., the Prinzmetal angina, which is characterized with spontaneous coronary spasms, and who ultimately suffered sudden cardiac death [85]. The clinicopathological obervations strongly suggested that the soluble vasoactive substances, such as histamine, prostaglandin D2, and leukotrienes C4 and D4 derived from the adventitial mast cells present in the atherosclerotic segments of the culprit coronary artery had caused the ultimately fatal spastic angina in this patient. Intriguingly, the cytoplasm of human mast cells contains numerous triglyceride-rich lipid droplets, which have been recently found to serve as a significant source of arachidonic acid for the production and release of eicosanoids upon mast cell activation [86].

As noted above, besides an IgE-dependent “anaphylactic degranulation” response, mast cells can be activated also by non-IgE-mediated activators with ensuing release of an individual mediator or release of a plethora of bioactive mediators as cocktails of varying compositions, and also constitutively secrete de novo synthesized cytokines and chemokines either singly or in varying combinations [73,74,87]. Obviously, such selective release of individual mediators escapes light microscopic observation, and therefore has remained unnoticed in the human arterial samples we have studied. Interestingly, cardiac events in humans may result from psychological or social stress, and, based on studies in restraint stressed mice, a role for the corticotropin-releasing hormone as a trigger of coronary mast cells in this context has been suggested [88].

Whether mast cell activation leading to the selective type of release of the granule contents, e.g., via piecemeal degranulation, also occurs in the inflamed human coronary arteries has not been determined. Actually, the piecemeal type degranulation has been identified in other settings which are also relevant to the mast cells in atherosclerotic human arteries [73]. They range from the chronic psychosocial stress [88,89] to presence of monocyte chemoattractant protein-1 (MCP-1 or CCL2) [90], to activators of Toll-like receptor 2 in mast cells [91], and to mast cell interactions with regulatory T cells [92]. Of note, identification of the inflammation-associated type of mast cell activation response (piecemeal degranulation) requires sophisticated ultrastructural analysis [93], and clearly remains a challenge for future studies when attempting to define the entire spectrum of mast cell activity in atherosclerotic lesions. Finally, whether release of cytokines and chemokines via constitutive exocytosis of secretory vesicles by lesional mast cells takes place has not been determined. The presence of TNF-α -containing cytoplasmic secretory granules in mast cells in the rupture-prone areas of human coronary atheromas supports the idea that this cytokine is released when a mast cell becomes activated to degranulate in these critical areas of atherosclerotic coronary arteries [94].

## 6. Mast Cells as Effector Cells in Atherogenesis

The sequence of the various steps in atherogenesis were outlined in the first part of this review, and here the potential modulating effects of mast cells on the individual steps will be briefly described and interpreted in the light of the presented scheme of atherogenesis. For a more extensive description of the processes, the reader is referred to a recent review in which both the activators and actions of mast cells in atherosclerotic cardiovascular disease, in cell culture systems, and particularly in mouse models of atherosclerosis are described in more detail [75]. In the said review, we describe a study, in which a comprehensive and versatile series of experiments in an atherosclerotic mouse model were performed in Leiden, the Netherlands by one of the two authors. In that particular study, carotid artery plaque formation was induced by local perivascular collar placement, as described earlier [95]. The fundamental advantage in the collar model used is a rapid induction of carotid atherosclerosis, and the possibility to locally activate and inhibit the adventitial mast cells present in the atherosclerotic arterial segment [11]. The experimental options then allow not only testing the various proposed hypotheses about the atherosclerosis-modulating roles of mast cells but also testing novel mast cell activating ligands and their inhibitors *in vivo*. In the following sections, some insights will be provided into the potential roles of mast cells in the initiation and progression of atherosclerosis. Such information has been gained from experimental systems involving *in vitro* incubations, cellular co-cultures, and mouse models of atherosclerosis, in which arterial mast cells are stimulated locally or in which mast cells are genetically absent.

### 6.1. Actions Related to Early Atherogenesis

The mast cells can contribute to the initiation of atherogenesis by accelerating infiltration of circulating LDL particles into the intimal layer of an atherosclerosis-susceptible arterial segment. By increasing the permeability of aortic endothelium to circulating LDL particles via endothelial histamine H1 receptor activation, histamine drives the formation of atherosclerosis in hypercholesterolemic mice [96]. Of note, although mast cells are major histamine stores in tissues, histamine can be synthesized also by endothelial cells and other types of cell, so preventing one to define a histamine-dependent effect automatically mast cell-specific. This restrictive specification applies to most mast cell-derived compounds, with the notable exception of the mast cell-specific compounds heparin and tryptase. Also, tryptase can break the endothelial barrier to LDL particles in a PAR-2-dependent fashion [97].

Activated mast cells secrete monocyte-attracting chemokines, and they can also activate the endothelial cells to express adhesion molecules, thereby inducing monocyte recruitment to the subendothelial space [98]. Moreover, cell culture experiments have shown that mast cell chymase specifically cleaves the carboxyl terminal part of the anti-inflammatory apoA-I component of the lipid-poor pre-beta HDL particles [99]. The C-terminally truncated apoA-I, again, was found to be unable to suppress the TNF-α-induced expression of adhesion molecules in human coronary artery endothelial cells. Thus, by secreting chemokines, TNF-α, and neutral proteases, subendothelially activated coronary mast cells may significantly contribute to monocyte recruitment to inflamed athero-prone coronary sites.

In the intimal space, the exocytosed heparin-containing mast cell granules may bind LDL particles via electrostatic interactions between granule heparin and the apoB-100 component of LDL [43]. Then the granule heparin-bound chymase proteolyzes the apoB-100, and renders the LDL particles unstable, whereupon the particles fuse to form larger particles, reminiscent of those found extracellularly in human atherosclerotic lesions. The fusion process of LDL particles on granule surface allows more LDL particles to bind to the granule, and so allows a heavier LDL cholesterol load per granule. The newly discovered proteolytic LDL aggregation/fusion model has served as a model for understanding of supersaturation of LDL binding to extracellular intimal proteoglycans with ensuing continuous accumulation of extracellularly located cholesterol in the evolving intimal lesions [14,100]. Mechanistically, also a novel pathway for foam cell formation was described, as it turned out that macrophages avidly phagocytose such LDL-coated mast cell granules, become filled with the cholesterol contained in the granule-bound LDL particles, and ultimately turn into foam cells [101,102]. With immunoelectron microscopic techniques, evidence was obtained that such “granule carrier pathway” may indeed operate *in vivo* in the human arterial intima [103].

Histamine released by the activated mast cells also increases endothelial permeability to the HDL particles [104]. Among the particles, the preβ-HDL particles are considered to be the most efficient ones for removal of cholesterol from the macrophage foam cells. However, this process can be blocked by proteolytic degradation of the extremely protease-sensitive preβ-HDL particles, which may become degraded by either the neutral protease tryptase and/or chymase released by an activated mast cell [105]. Thus, activated mast cells contribute to foam cell formation by both increasing uptake of the cholesterol-containing LDL particles and by decreasing the ability of HDL particles to induce efflux of the accumulating intracellular cholesterol.

### 6.2. Actions Related to Advancing and Terminal Atherogenesis

The continuous influx of LDL-cholesterol into macrophages with ensuing foam cell formation, a process to which phagocytosis of LDL-granule complexes and insufficient efflux of cholesterol from the formed macrophage foam cells to mast cell-modified HDL particles may jointly contribute, appears to immobilize the cells in the lesions, and, ultimately to render them susceptible to death [3]. Moreover, histamine released by activated mast cells may induce apoptotic death of macrophages, and thereby contribute to the formation of a necrotic lipid core and plaque growth [11].

As the plaque grows and obstructs the arterial lumen, the blood flow becomes turbulent and the endothelial cells become dysfunctional and may also detach. Of note, depending on the phenotype, the subendothelially located mast cells may be activated to release tryptase, or both tryptase and chymase, which then degrade the endothelial basement membrane with ensuing physical detachment of the endothelial cells [106]. Moreover, by losing contact with the basement membrane, the endothelial cells lose their outside-in survival signaling and become apoptotic, a process facilitated by release of TNF-α by the mast cells [107]. Recently, endothelial erosion has been proposed to ensue partly due to the activity of circulating neutrophils, which could be recruited to the vulnerable areas partly via mast cell-derived IL-8 [108,109]. Thus, the endothelial cells covering an advanced atherosclerotic plaque are under threat of being attacked from both the abluminal and the luminal side, i.e., by activated mast cells and activated neutrophils, respectively. Of note, however, mature mast cells are located under the endothelium only, while the neutrophils are likely to attack the endothelial cells mainly from the luminal side, and to accelerate the growth of the forming arterial thrombus after the detachment of the endothelial cells [108].

Thinning of the fibrous cap of an atherosclerotic plaque results from gradual loss of intact collagen fibers in the cap, which, again, is a composite of reduced formation and increased degradation of the fibers, the former resulting from senescence and apoptotic death of the collagen-producing smooth muscle cells, and the latter being caused by overexpression and activation of collagenolytic enzymes, notably the matrix metalloproteinases (MMPs) [110,111]. Chymase released from mast cells can induce apoptotic death of smooth muscle cells by degrading fibronectin of their pericellular matrix, and so blocking the outside-in survival signaling necessary for their survival [112]. Moreover, chymase inhibits collagen synthesis in smooth muscle cells by both TGFβ-dependent and -independent mechanisms [113]. Regarding the MMP-dependent collagen catabolism, chymase and tryptase can activate the inactive proforms of MMP-1 and MMP-3, respectively [114,115]. Importantly, Johnson and coworkers demonstrated in the vulnerable shoulder regions of human atherosclerotic carotid plaques increased MMP-1 and MMP-3 expression, which was associated with an increased MMP net activity in areas where also increased numbers of degranulated mast cells were present [116]. Taken together, by releasing tryptase and chymase, activated mast cells in advanced atherosclerotic lesions appear to significantly contribute to the lesion-weakening proteolytic events directly, and also indirectly by their MMP-activating capabilities.

Since the arterial intima lacks capillaries, hypoxia easily develops in the deep intima, and is further exacerbated by the additional increase in intimal thickness taking place during atherogenesis [117,118]. Particularly, the formation of a necrotic lipid core prevents oxygen diffusion into the deep intima. As a response, the deep intimal layers become neovascularized when the pre-existing medial branches of adventitial *vasa vasorum* grow into the lesion and form a microvascular network [119]. Obviously, capillarization of the deep hypoxic areas tends to prevent hypoxic cellular dysfunction and death, and thereby tends to stabilize an atherosclerotic plaque. However, at the same time the neovascularization entails the risk of intraplaque hemorrhage, which will destabilize the plaque and thereby increase its vulnerability to rupture [119,120].

The mast cells are proangiogenic cells par excellence, and their role in tumor growth-related angiogenesis has been studied in depth in cancer biology [121]. Interestingly, mast cells survive hypoxia and even become activated in response to hypoxia [122]. Accordingly, mast cells possess the capacity to contribute to plaque neovascularization by releasing a variety of angiogenic mediators, not only by releasing the classical and ubiquitous angiogenic growth factors, notably the Vascular endothelial growth factor (VEGF) and the Basic fibroblast growth factor (bFGF), but also mast cell-specific substances, such as heparin and the major granule proteases tryptase and chymase [123,124,125].

The above dichotomy regarding neovascularization—initially good and then bad—also applies to mast cells in advanced atherosclerotic lesions [120,126,127]. Thus, we could observe mast cells in close proximity of intact coronary microvessels, and also in areas with intraplaque hemorrhages. As protease-secreting cells, the subendothelial mast cells have the potential to degrade the endothelial basement membrane of the already formed microvessels by the mechanisms described above for the plaque-covering endothelium [106]. Most importantly, however, in the deep intimal areas the endothelial basement membrane of the neovascular sprouts is poorly developed, which must render the microvessels exceptionally sensitive to protease-triggered leakage with ensuing intimal hemorrhage and destabilization of the plaque [128,129]. This may also apply to advanced human carotid atherosclerotic plaques, in which activated mast cells are present [130,131,132]. Indeed, regarding the clinical relevance of mast cell-induced intraplaque neovascularization, Willems and coworkers found that in advanced human carotid atherosclerotic plaques mast cells are present in the deep neovascularized areas, and that their numbers strongly associate with microvessel density in patients with symptom-causing carotid plaques, i.e., plaques with erosion or rupture [133]. Moreover, high numbers of mast cells in the neovascularized areas also predicted the occurrence of future cardiovascular events, possibly reflecting an advanced stage of the disease in other atherosclerosis-susceptible arterial segments, where mast cells may also have contributed to disease progression.

Finally, could we measure the activity of mast cells of a given prespecified tissue in a clinical setting, for example in the emergency room where a patient is examined and managed for an acute coronary syndrome? Unfortunately, it is not possible at present. Yet, when compared with macrophages and T cells, we can measure at least the plasma level of a molecule, the tryptase, which is derived from mast cells and from mast cells only. However, determination of the level of circulating tryptase reflects both the constitutive mast cell-derived and the mast cell activation-dependent release of tryptase, and, moreover, it reflects the amount tryptase released by the totality of mast cells in the human body [134]. When compared with the total number of mast cells in the human body, the number of mast cells in the coronary arteries must be extremely small—maybe one per-mille or even much less. Thus, it is unlikely that their basal production or activation-triggered release would lead to measurable changes in the level of the circulating tryptase. Yet, an interesting observation has been made, which showed that in patients with acute coronary syndromes with or without an ST-elevation, measurement of serum tryptase level at admission improved the risk stratification regarding recurrent myocardial infarction within 2 years [135]. Since atherosclerosis is a systemic disease potentially affecting all susceptible arterial segments, we can surmise that, in the cited study, the significantly increased serum tryptase levels in the patients with severe coronary atherosclerosis actually reflected the aggregate quantity of tryptase derived from the totality of mast cells present in all severely inflamed atherosclerotic lesions of the body. 

## 7. Concluding Remarks: Allergic Atherosclerosis—Does One Exist?

Activated mast cells are present in human atherosclerotic lesions throughout the lesion development—i.e., from start to end. Despite the presence of these “allergy cells” in the lesions, there is little or no evidence that the chronic low-degree inflammation in the atherosclerotic plaques would possess an allergic component, with the notable exception of the Kounis syndrome. Yet, an association of elevated serum IgE antibody levels with the severity of coronary artery disease and with acute coronary events has been observed [136]. However, it is not known whether the mast cells in the human epicardial coronary arteries carry antigen-specific IgE bound to their surfaces, which is a central requirement for acute allergic reactions. On the other hand, mast cells isolated from human myocardium have been found to harbor IgE-receptor-bound IgE antibodies on their surfaces [137]. Interestingly, however, mere presence of IgE on mast cells may be sufficient for their IgE-mediated activation without the classical IgE-specific antigen (allergen)-triggered crosslinking of two receptor-bound IgE antibodies being necessary. Indeed, there is an alternative mechanism of IgE-mediated activation available for the human myocardial mast cells. Thus, such mast cells can be activated by endogenous and exogenous superantigenic (bacterial or viral) stimuli when the immunoglobulin superantigens interact with different regions of the IgE antibodies bound to their high-affinity receptors on mast cell surface [70]. Moreover, there is evidence that some IgE preparations, by themselves, can activate mouse and human mast cells to induce selective release of cytokines, so reflecting structural and functional heterogeneity among human IgE antibodies [138]. Whether the above-described mast cell activation by IgE in the absence of antigen would also occur in human coronary arteries remains to be determined. Nevertheless, the unequivocal demonstration of mast cell degranulation in atherosclerotic human coronary arteries, which strongly depends on the severity of disease, unequivocally demonstrates the presence of “anaphylactic” activation of the mast cells, whatever the nature of the actual stimulus would be. At present, the top candidates for being the actual degranulating agents of human intimal and the adventitial mast cells are the anaphylatoxins of the complement system and the neuropeptides, respectively [81,82,84]. 

Answers to the above-mentioned questions about the actual stimulators of mast cells in human atherosclerotic lesions are now sprouting. Thus, in a very recent paper Kritikou and co-workers elegantly describe a flow cytometry-based characterization of mast cells in plaque samples collected after femoral or carotid endarterectomy surgery [139] They found that most of the intraplaque mast cells were activated, and that most of the activated mast cells had bound IgE fragments on their surfaces, while another mast cells showed IgE-independent activation. Thus, as the authors suggest that IgE-dependent activation and actions of mast cells in advanced human atherosclerosis offer potential for therapeutic interventions. However, when considering the emerging understanding of the functional heterogeneity of the IgE molecules, the identity of the actual IgE-dependent mast cell-activating ligands—whether (super)antigenic or non-antigenic—needs to be determined. 

It is important to state that atherosclerosis is not an infectious disease *per se*. Yet, gingival infections and variations in the gut microbiota pose an increased cardiovascular risk [140,141]. Thus, immunologically active molecules, such as lipopolysaccharides, or even whole bacteria may escape the oral, intestinal, or respiratory mucosal surfaces, and reach the atherosclerotic lesions and locally accelerate the lesion development [142,143,144,145]. In this regard, the novel observation by Gupta and coworkers demonstrating that host defense peptides and the lipopolysaccharide derived from porphyromonas gingivalis can activate mast cells to degranulate is of extraordinary interest [146].

**Figure 2:**
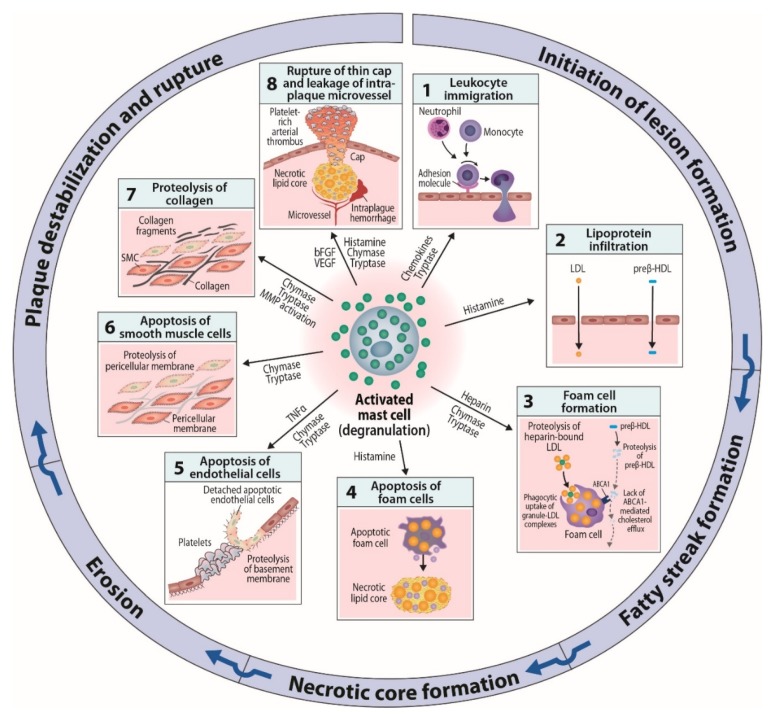
Potential effects of intimal mast cells on initiation and progression of human atherosclerotic lesions. **I. Initiation of lesion formation**. (**1**). Activated subendothelial mast cells contribute to leukocyte recruitment by releasing chemokines, and by also releasing tryptase and TNFα which enhance adhesion molecule expression on endothelial cells. (**2**). Activated mast cells release vasoactive substances, notably histamine, which increase endothelial permeability for low-density lipoprotein (LDL) and high-density lipoprotein (HDL) particles. **II. Fatty streak formation**. (**3**). Subendothelially, LDL particles bind to the heparin component of exocytosed mast cell granules, after which granule chymase proteolyzes the particles and renders them unstable and susceptible to fuse with each other. When macrophages phagocytose such complexes composed of granule-bound fused LDL particles, they become filled with LDL-derived cholesterol and are converted to foam cells filled with cholesteryl ester-containing lipid droplets. The granule neutral proteases chymase and tryptase degrade preβ-HDL particles, which thereby lose their ability to interact with the ABCA1 transporter on macrophage foam cells and to accept cholesterol from the foam cells. **III. Necrotic core formation**. (**4**). Mast cell-derived histamine is able to induce macrophage apoptosis. When a macrophage foam cell dies, the generated cellular debris and the liberated lipid droplets contribute to the formation of an extracellular necrotic lipid core. The formation of a core is the hallmark of conversion of an early fatty streak lesion into an advanced atherosclerotic plaque, which consists of a core and a collagen cap. The cap separates the strongly thrombogenic core from the circulating blood. **IV Erosion**. (**5**). Release of tryptase and/or chymase by activated subendothelial mast cells induce degradation of endothelial basement membrane with ensuing apoptosis and detachment of the involved endothelial cells. Mast cell-derived TNFα contributes to endothelial apoptosis, while mast cell-derived heparin tends to attenuate the growth of the forming platelet-rich arterial thrombus at the site of erosion. **V. Plaque destabilization and rupture.** (**6**). Activated mast cells in the collagen cap release chymase, which degrades the pericellular matrix of smooth muscle cells with ensuing apoptotic death of the cells due to loss of outside-in survival signaling. Loss of the collagen-producing smooth muscle cells reduces net collagen formation in the cap, and so weakens it. (**7**). Release chymase and tryptase by mast cells in the collagen cap locally activates extracellularly located proforms of matrix metalloproteinases, and so triggers collagen degradation which further weakens the cap and destabilizes the plaque. (**8**). Mast cell-derived angiogenic factors induce growth of microvessels into the hypoxic regions of the otherwise avascular plaque. Mast cell-derived tryptase and chymase, again, may degrade the fragile microvessel walls, and so trigger microvascular hemorrhage which contributes to plaque instability. Together, cap thinning and intraplaque hemorrhages render the plaque susceptible to rupture with ensuing formation of a large lumen-occluding coronary thrombus.

In summary, experimental evidences derived from cell culture systems and animal models have provided multiple clues to the mechanisms by which the mediators released from activated mast cells may affect the development of atherosclerotic lesions at various stages of the disease (Figure 2). To learn about possible mast cell subsets endowed with specific repertoires of receptors and effector functions in human atherosclerotic lesions, their isolation and functional testing at the single-cell level is required. Such knowledge will open up opportunities to design sensible experiments in which we selectively or jointly modify the proatherogenic functions, and possibly also improve anti-atherogenic functions of mast cells in the evolving atherosclerotic lesions.

The emerging challenge now is to find homeostatic and healing functions for the coronary mast cells, like those found for the myocardial mast cells. The ability of macromolecular heparin proteoglycans derived from rat peritoneal mast cells to attenuate the growth of a platelet-rich arterial thrombus in experimental systems can be considered as a model of such homeostatic self-correcting functions of mast cells [147,148]. Unless we find such potentially life-saving functions for the mast cells present in human atherosclerotic lesions, we have to ask ourselves, whether we are failing to understand the very essence of these cells evolved as protective sentinel cells about 500 million years ago, or whether the mast cells in atherosclerotic lesions have arrived at a wrong address.
